# Revolutionizing Treatment for Chronic Subdural Hematoma: Promising Outcomes With Middle Meningeal Artery Embolization

**DOI:** 10.7759/cureus.39733

**Published:** 2023-05-30

**Authors:** Ali Msheik, Teddy Gerges, Zeinab Al Mokdad, Daniel Abbass, Assaad Mohanna, Ahmad Aoude

**Affiliations:** 1 Neurological Surgery, Lebanese University Faculty of Medicine, Hadath, LBN; 2 Anesthesia, Winchester Anesthesia Associates, Boston, USA; 3 Public Health, Lebanese University Faculty of Medicine, Beirut, LBN; 4 Neurological Surgery, Al Rassoul Al-Aazam Hospital, Beirut, LBN; 5 Radiology, Bahman Hospital, Beirut, LBN; 6 Neurosurgery, Al Rassoul Al-Aazam Hospital, Beirut, LBN

**Keywords:** brain trauma indicator, new oral anticoagulants ( noacs), neuro-surgery, mma embolization, chronic subdural hematoma (csdh)

## Abstract

Chronic subdural hematoma (cSDH) is a common condition that typically requires surgical intervention. Middle meningeal artery embolization (MMAE) has emerged as a potential alternative treatment option, although the choice of embolization material remains a topic of debate. In this case series, we report on the outcomes of 10 patients with cSDH who underwent MMAE. Most patients experienced symptom relief and a significant reduction in cSDH size post-procedure. Despite the presence of comorbidities and risk factors, most patients had positive outcomes following MMAE treatment. Only one patient required surgical intervention after the MMAE procedure due to the progression of symptoms, while MMAE successfully prevented recurrence in most patients. Our findings suggest that MMAE can be a promising treatment option for selected patients with cSDH. However, further studies are needed to compare the efficacy and safety of different embolization materials in MMAE procedures for cSDHs.

## Introduction

Chronic subdural hematomas (cSDH) are collections of liquefied blood in the subdural space, enclosed by an outer membrane that varies in thickness from 1 to 10 millimeters and an inner membrane [1]. cSDHs occur bilaterally in 19% of cases. They are associated with neoangiogenesis from dural capillaries and marginal cell proliferation in the dural layer, leading to the growth of the outer cSDH layer and increased bleeding [2]. Tearing of bridging veins that traverse the dural cell layer is the most frequent cause of cSDHs [3]. Careful assessment of each patient is necessary before initiating cSDH medication, and surgical intervention may be required depending on the patient's symptoms [4]. Most patients with cSDH are elderly and have pulmonary conditions, cardiac inadequacy, renal impairment, advanced age, and are taking oral anticoagulants (OACs) and antiplatelet medication [5]. Cerebral decompression and surgical evacuation are the main therapies for symptomatic cSDHs, with postoperative adjuvant treatments such as corticosteroids, angiotensin-converting enzyme (ACE) inhibitors, and tranexamic acid being underexamined in the literature [6]. Administration of corticosteroids is associated with decreased recurrence rates, while the efficacy of the other two treatments requires further investigation [7]. Recently, middle meningeal artery embolization (MMAE) has been proposed as a treatment plan to minimize recurrence and prevent additional rebleeding [8]. The anterior and posterior branches of the MMA supply the dural layers and are hypothesized to anastomose with neovascular arteries, leading to cSDH recurrence if these anastomoses rebleed. Embolization of the MMA branches near the site of the cSDH is believed to reduce bleeding and prevent recurrence [9]. An endovascular technique is required for embolization through the middle meningeal artery in proximity to the recurrent cSDH. After guided travel through the femoral artery, the aorta, and either the left or right carotid artery, the MMA branches off from the maxillary branch of the external carotid artery. An embolization agent, such as N-butyl-2-cyanoacrylate, is typically used to embolize the targeted portion. The subsequent stagnation of contrast confirms the success of the embolization [10]. The utilization of MMAE necessitates studies that report on this method's results, especially considering that surgical evacuation has been extensively documented for over 60 years in the literature.

The researchers involved in this prospective case series hypothesized about the results of MMAE and the findings associated with its application. This study aims to evaluate the use of MMAE, drawing on an algorithm devised from previous and recent systematic reviews of MMAE for the treatment of cSDHs [11].

## Materials and methods

**Figure 1:**
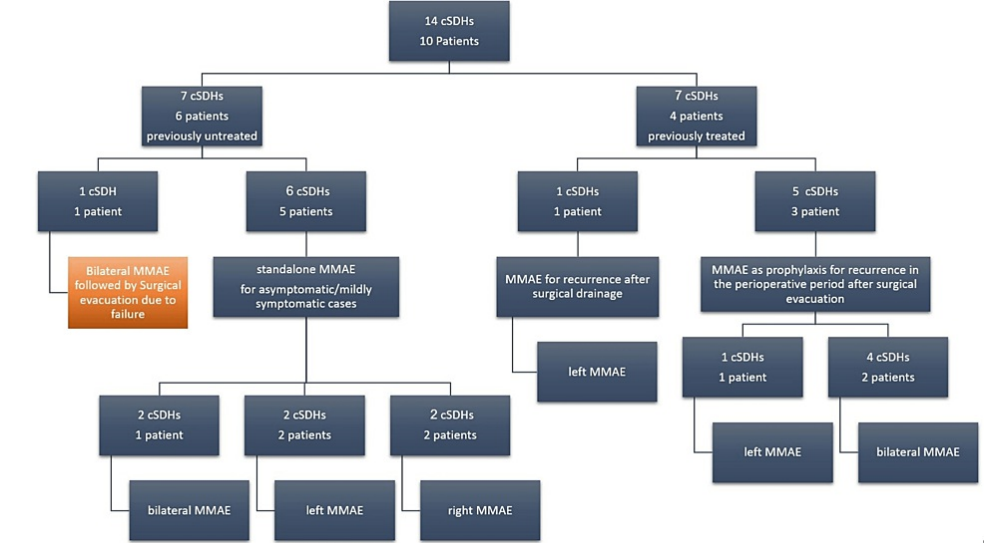
Distribution of patients according to findings and procedures. *Patient diagnosed with bilateral cSDHs after ventricular-peritoneal shunting due to cranial penetrating injury-provoked hydrocephalus. MMAE: Middle meningeal artery embolization; cSDH: Chronic subdural hematoma.

This is a prospective observational study involving 10 patients who received MMAE for the treatment of cSDHs between January 2021 and February 2023 (Figure 1). Ethical clearance was obtained from the relevant committee with approval number 17/2021, and all patients provided informed consent before the procedure. The inclusion criteria included radiological evidence of a cSDH with minimal neurological aberrations in high-risk patients without the need for urgent decompression, as well as patients with recurrent cSDHs. Eligible patients were diagnosed with cSDH based on cranial CT findings. They received MMAE for various indications, including standalone treatment for incident or mildly symptomatic cSDHs, recurrence of previously evacuated cSDHs, and as a prophylactic measure immediately following a surgical evacuation in high-risk patients [11]. The exclusion criteria included mixed-density SDHs with >50% acute component, neurological deficits suggestive of brainstem or cranial nerve compression, and intolerance to contrast material. All MMAE procedures were performed under general anesthesia in the angiography unit. A 5-Fr guiding sheath was inserted into the right femoral artery and guided into the common carotid artery under the administration of general anesthesia. Systemic heparinization was administered, and a 3.4-Fr distal access catheter was introduced into the external carotid artery. The distal access catheter was guided near the foramen spinosum, and a 1.3-Fr flow-guided microcatheter was navigated into the MMA using a 0.010-inch guidewire. The embolization procedure targeted the anterior and posterior convexity branches. For the anterior branch, MMA angiography was performed to confirm the absence of anastomosis with the ophthalmic artery. The microcatheter was positioned above the orbital roof to minimize the risk of ophthalmic artery embolization. Immediately after the embolization procedure, high-resolution CT (HRCT) scans were conducted to examine the distribution of the embolic agent. Subsequently, the patients were kept under observation in the general ward and evaluated for any neurological symptoms. The following day, CT scans were performed, and depending on their condition, the patients were either discharged to their homes or transferred to a rehabilitation hospital. Additional postoperative CT scans were conducted at one and three months after the procedure.

Demographic data, findings, and follow-up changes were recorded. The follow-up period for each patient was two months, with routine imaging and clinical check-ups conducted at two and six weeks after the procedure. We identified a set of established comorbidities and risk factors for MMAE (major adverse cardiovascular events). These variables of interest include age, sex, essential/primary hypertension, type 2 diabetes mellitus, nicotine dependence, overweight/obesity, chronic kidney disease (CKD), atrial fibrillation/flutter, asthma, ischemic heart diseases within the past ten years (ICDs I20-I25), other heart diseases within the past ten years (ICDs I30-I52), usage of antiplatelet medications (such as aspirin and clopidogrel), anticoagulation medications (such as apixaban, heparin, rivaroxaban, and warfarin), and end-stage renal disease.

Outcome analysis

Primary clinical outcomes were defined as the proportion of patients who experienced the specific outcome of interest. The outcomes studied included mortality, repeated MMAE, craniectomy or craniotomy procedures, and neurological deficits. The analysis of outcome data was conducted using non-contrast brain CT scans to evaluate complete, partial, same, and worse outcomes following MMAE (major adverse cardiovascular events), as well as procedural complications. The resolution was categorized as complete in cases where the hematoma volume was less than 10 ml. The partial resolution was defined as a subdural hematoma (SDH) volume exceeding 10 ml [12].

## Results

A total of 10 patients with cSDHs were included in this case series, with an average age of 63.4±15.99 years (Table 1). Out of the 10 patients, six were male. The most common presenting symptom was headache (eight patients), followed by hemiparesis and altered consciousness (three patients each). At presentation, seven patients had multiple symptoms, including dysarthria, ataxia, and dizziness. Five patients had hypertension, and three had type II diabetes mellitus. One patient had hydrocephalus due to a previous penetrating cranial injury and had been previously treated with ventricular-peritoneal shunting, which led to the development of a bilateral cSDH. Among the 10 patients, six were taking oral anticoagulants, with four using acetylsalicylic acid, two using clopidogrel, one using dabigatran, and one using acenocoumarol. Only two patients were smokers.

**Table 1:** Clinical characteristics and risk factors of patients.

Patient characteristics			Frequency, n=10
Presenting Symptoms	Headache		8
	Hemiparesis		3
	Altered consciousness		3
	Dysarthria		1
	Ataxia		1
	Dizziness		1
Comorbidities	Epilepsy		1
	Hypertension		5
	Diabetes Mellitus Type II		3
	Acute myelogenous Leukemia		1
	Coronary artery disease with a history of percutaneous transluminal venous coronary angiography		1
	Hydrocephalus		1
	Mitral Valve replacement		1
	End-stage renal disease		1
Risk Factors	Smoking		2
	Oral anticoagulants	Acetylsalicylic acid	4
		Acenocoumarol	1
		Clopidogrel	2
		Dabigatran	1
Gender	Female		4
	Male		6
Age (years) average ±SD^*^			63.4±15.99
History of head trauma			6

**Figure 2:**
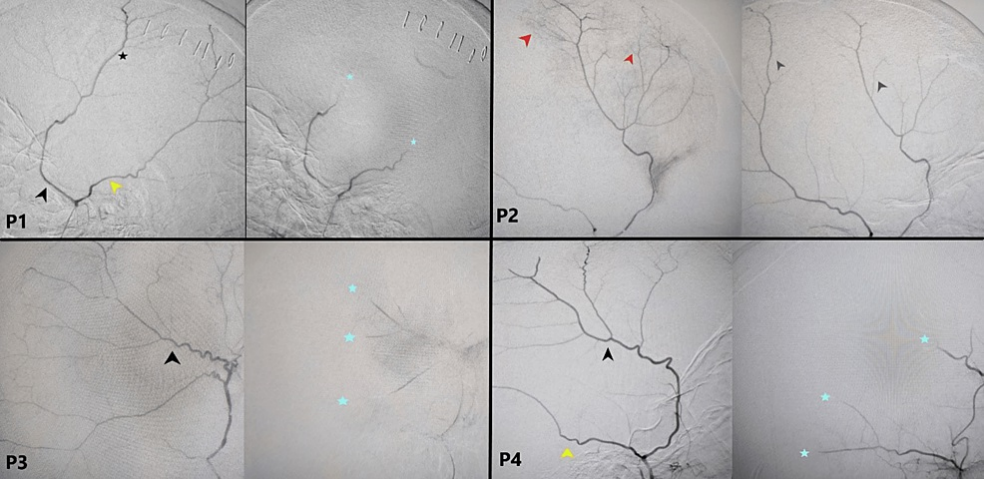
Sample digital subtraction angiographs (DSA) of four patients. Right-sided images: Pre-MMAE; Left-sided images: Post-MMAE; p: patient; Black arrowhead: Anterior MMA; Yellow arrowhead: Posterior MMA; Red arrowhead: terminal Blush pre-MMAE; Grey arrowhead: terminal Blush post-MMAE; Black Star: MMA before embolization; Blue Star: MMA post embolization. Source: Al Rassoul Al-Aazam Radiology Department. Permission was obtained.

MMAE using Onyx particles was applied to all patients, and the results of the digital subtraction angiography are illustrated in Figure 2.

**Figure 3:**
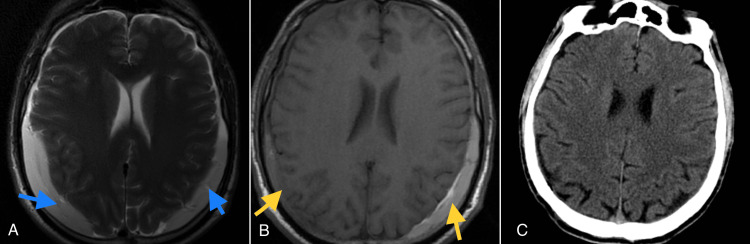
Evolution of a bilateral cSDH before and after MMAE. A: Cranial MRI showing bilateral cSDHs at presentation; B: Cranial MRI three weeks Post-MMAE; C: Cranial CT scan six weeks post-MMAE. Blue arrows: large cSDH at presentation; Yellow arrows: diminished size cSDHS three weeks post-MMAE. cSDH: Chronic subdural hematomas; MMAE: Middle meningeal artery embolization.

All patients resumed follow-up with a minimum of six weeks after the procedure. Only one patient required surgery one week after the MMAE procedure due to the progression of hemiparesis and altered consciousness, which warranted surgical decompression. No surgical intervention was necessary among the patients to whom MMAE was applied as a standalone procedure (5/10). One patient, who underwent MMAE for recurrence after the first surgical drainage, and three patients who received MMAE as prophylaxis for recurrence after the first surgical drainage, did not require surgical evacuation thereafter. All patients were symptom-free three weeks after the MMAE procedure. Six out of ten patients showed more than a 50% reduction in the size of the cSDHs two weeks post-MMAE, while nine out of ten patients showed more than a 50% reduction six weeks post-MMAE. Figure 3 illustrates the evolution of a bilateral cSDH. One patient with congestive heart failure and end-stage renal disease required urgent surgical decompression of a bilateral cSDH despite undergoing MMAE 10 days before the surgical intervention due to acute deterioration and left-sided hemiparesis and hemiplegia. The patient stabilized after the surgical decompression, and two days after the procedure, the patient's condition improved back to the preoperative status. The patient passed away two months after discharge due to cardiac and renal complications unrelated to the procedure (Table 2). No procedure-related complications were recorded.

**Table 2:** Post-MMAE findings. cSDH: Chronic subdural hematoma; MMAE: Middle meningeal artery embolization.

Post-procedure findings	Frequency, n=10
Follow-up >6 weeks available	10
Required surgery after MMAE	1
Avoidance of first surgery	5
Avoidance of surgery for recurrence	4
cSDHs size reduction >50% in 2 weeks post-MMAE	6
Complications due to the procedure	0

## Discussion

The results of the 10 patients treated with MMAE for chronic subdural hematoma (cSDH) demonstrate promising outcomes, with the majority of patients experiencing symptom relief and a significant reduction in cSDH size post-procedure. Among the patients, 8 out of 10 presented with headaches, which is a common symptom of cSDH and aligns with previous literature [13]. Hemiparesis and altered consciousness were also frequent symptoms, consistent with previous studies that have shown these symptoms to occur in patients with larger or more severe cSDH [14]. It is notable that the patients in this study had various comorbidities, with hypertension being the most common (5 out of 10), followed by type II diabetes mellitus (3 out of 10). Furthermore, several patients were on oral anticoagulants, which is a known risk factor for cSDH [15]. Despite these comorbidities and risk factors, the majority of patients in this study achieved positive outcomes following MMAE treatment.

**Figure 4:**
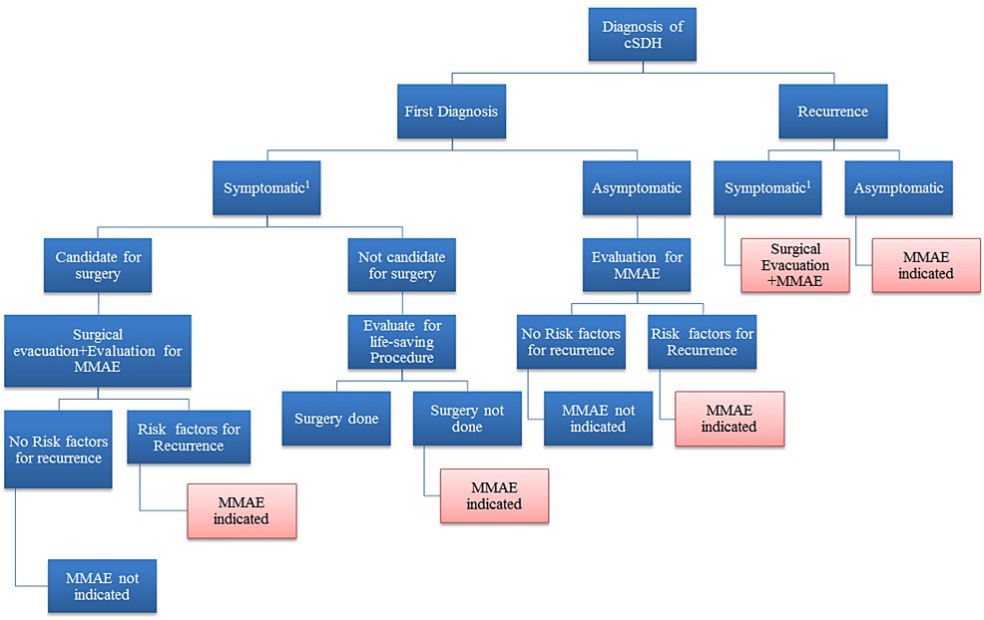
Algorithm and middle meningeal artery embolization indication advised by the authors. ^1^Neurological deficit; cerebral midline shift evident on imaging. Blue dashed arrow: Management continues similarly. cSDH: Chronic subdural hematoma; MMAE: Middle meningeal artery embolization. Permission was obtained from the authors.

Among the 10 patients, only one required surgical intervention after the MMAE procedure due to the progression of hemiparesis and altered consciousness. This finding is consistent with previous studies that have shown MMAE to be an effective standalone treatment for cSDH in selected patients [16]. Additionally, MMAE was successful in preventing recurrence in the majority of patients, with only one patient requiring surgical evacuation after MMAE. A recent literature review provided insights into the indications of MMAE and the frequency of its application for each indication (Figure 4) [11]. These findings align with previous studies that have demonstrated the efficacy of MMAE in preventing the recurrence of cSDH [17]. It is important to note that one patient in our study passed away two months after undergoing MMAE due to cardiac and renal complications unrelated to the procedure. While this outcome is tragic, it is crucial to acknowledge that MMAE is generally considered a safe procedure with low rates of complications [16].

In terms of the type of material used in the embolization procedure, our study did not specifically investigate this aspect. However, previous studies have reported the use of various materials, including Gelfoam, Onyx, and n-Butyl cyanoacrylate, in MMAE procedures for cSDHs. A study by Cho WS et al. [18] compared the use of Gelfoam and Onyx in MMAE procedures and reported similar efficacy and safety profiles for both materials. Another study by Kwon et al. [19] reported the use of n-Butyl cyanoacrylate in MMAE procedures, which demonstrated favorable outcomes with no significant complications.

Overall, the results of our study suggest that MMAE is a viable alternative to surgical evacuation for the treatment of cSDHs, with favorable outcomes and a low risk of complications. The choice of embolization material may depend on various factors, such as availability, cost, and individual patient characteristics. However, further studies are needed to compare the efficacy and safety of different embolization materials in MMAE procedures for cSDHs. MMAE shows promise as a treatment option for selected patients with cSDH. However, it is important to note that this was a small prospective study with a limited follow-up period. Further studies with larger sample sizes and longer follow-up periods are needed to confirm these findings and assess the long-term efficacy and safety of MMAE for cSDH.

## Conclusions

This prospective study suggests that MMAE may be a promising alternative to surgical evacuation for the treatment of cSDHs, offering potential benefits such as the avoidance of surgery and reduction in cSDH size. The absence of complications in our series contributes to the growing body of literature supporting the safety and efficacy of MMAE. Further studies are needed to compare the outcomes of MMAE with those of surgical evacuation and to investigate the use of different embolization materials in MMAE procedures. However, based on our results, MMAE appears to be a viable treatment option for cSDHs in selected patients, particularly those who are poor surgical candidates or prefer a less invasive approach.
